# Exploring the immune interactions between *Oncomelania hupensis* and *Schistosoma japonicum*, with a cross-comparison of immunological research progress in other intermediate host snails

**DOI:** 10.1186/s13071-023-06011-9

**Published:** 2023-12-13

**Authors:** Hongyu Li, Yihan Chen, Yunhuan Zhu, Yilu Feng, Yuncheng Qian, Xiaoyu Ye, Jiatong Xu, Hanyu Yang, Jiawei Yu, Jingyu Chen, Keda Chen

**Affiliations:** 1 Shulan International Medical College, Zhejiang Shuren University, Hangzhou, China; 2 Ocean College, Beibu Gulf University, Qinzhou, China

**Keywords:** *Oncomelania hupensis*, *Biomphalaria glabrata*, Macrophage migration inhibitory factor (MIF), Toll-like receptors (TLRs), Thioredoxin (Trx)

## Abstract

Schistosomiasis, the second largest parasitic disease in the world after malaria, poses a significant threat to human health and causes public health issues. The disease primarily affects populations in economically underdeveloped tropical regions, earning it the title of “neglected tropical disease”. Schistosomiasis is difficult to eradicate globally if medication alone is used. One of the essential elements of thorough schistosomiasis prevention and control is the management and disruption of the life cycle of intermediate host snails. The key approach to controlling the transmission of schistosomiasis is to control the intermediate hosts of the schistosome to disrupt its life cycle. We believe that approaching it from the perspective of the intermediate host’s immunity could be an environmentally friendly and potentially effective method. Currently, globally significant intermediate host snails for schistosomes include *Oncomelania hupensis*, *Biomphalaria glabrata*, and *Bulinus truncatus*. The immune interaction research between *B. glabrata* and *Schistosoma mansoni* has a history of several decades, and the complete genome sequencing of both *B. glabrata* and *B. truncatus* has been accomplished. We have summarized the immune-related factors and research progress primarily studied in *B. glabrata* and *B. truncatus* and compared them with several humoral immune factors that *O. hupensis* research focuses on: macrophage migration inhibitory factor (MIF), Toll-like receptors (TLRs), and thioredoxin (Trx). We believe that continued exploration of the immune interactions between *O. hupensis* and *Schistosoma japonicum* is valuable. This comparative analysis can provide some direction and clues for further in-depth research. Comparative immunological studies between them not only expand our understanding of the immune defense responses of snails that act as intermediaries for schistosomes but also facilitate the development of more comprehensive and integrated strategies for schistosomiasis prevention and control. Furthermore, it offers an excellent opportunity to study the immune system of gastropods and their co-evolution with pathogenic organisms.

**Graphical Abstract:**

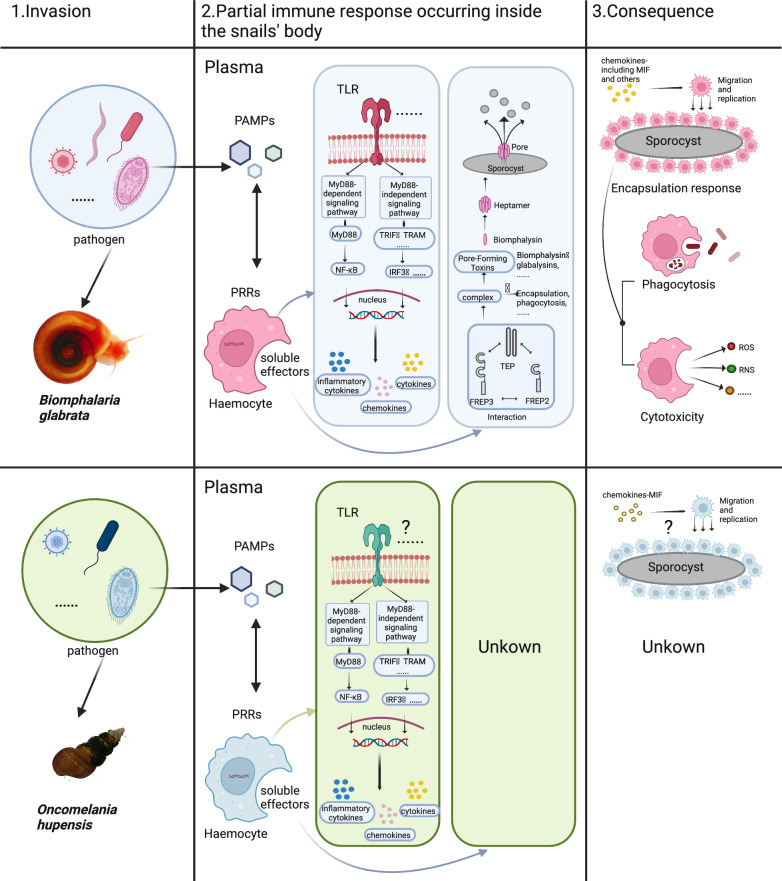

## Background

Schistosomiasis is a parasitic disease caused by an infestation of *Schistosoma*, which is a major public health problem in tropical and subtropical regions [1]. According to the World Health Organization's 2019 statistics, over 200 million people in more than 70 countries and regions in Asia, Africa, and Latin America are affected. The majority of patients are located in economically underdeveloped areas of Africa with low-income levels, which is why schistosomiasis is often referred to as a “neglected tropical disease” [2]. There are three main species of schistosomiasis parasites that affect humans: *Schistosoma mansoni*, *S. haematobium*, and *S. japonicum* [3]. *Schistosoma mansoni* is mainly distributed in tropical and subtropical regions of Africa and South America. *Schistosoma haematobium* is mainly distributed in Africa and the Middle East [4]. *Schistosoma japonicum* is mainly distributed in East Asia, including China, Japan, South Korea, and Southeast Asian countries [4]. Notably, the distribution of these schistosomiasis diseases is not completely fixed and may change over time and with conditions, influenced by factors such as climate, environment, and population migration [5].

**Fig. 1:**
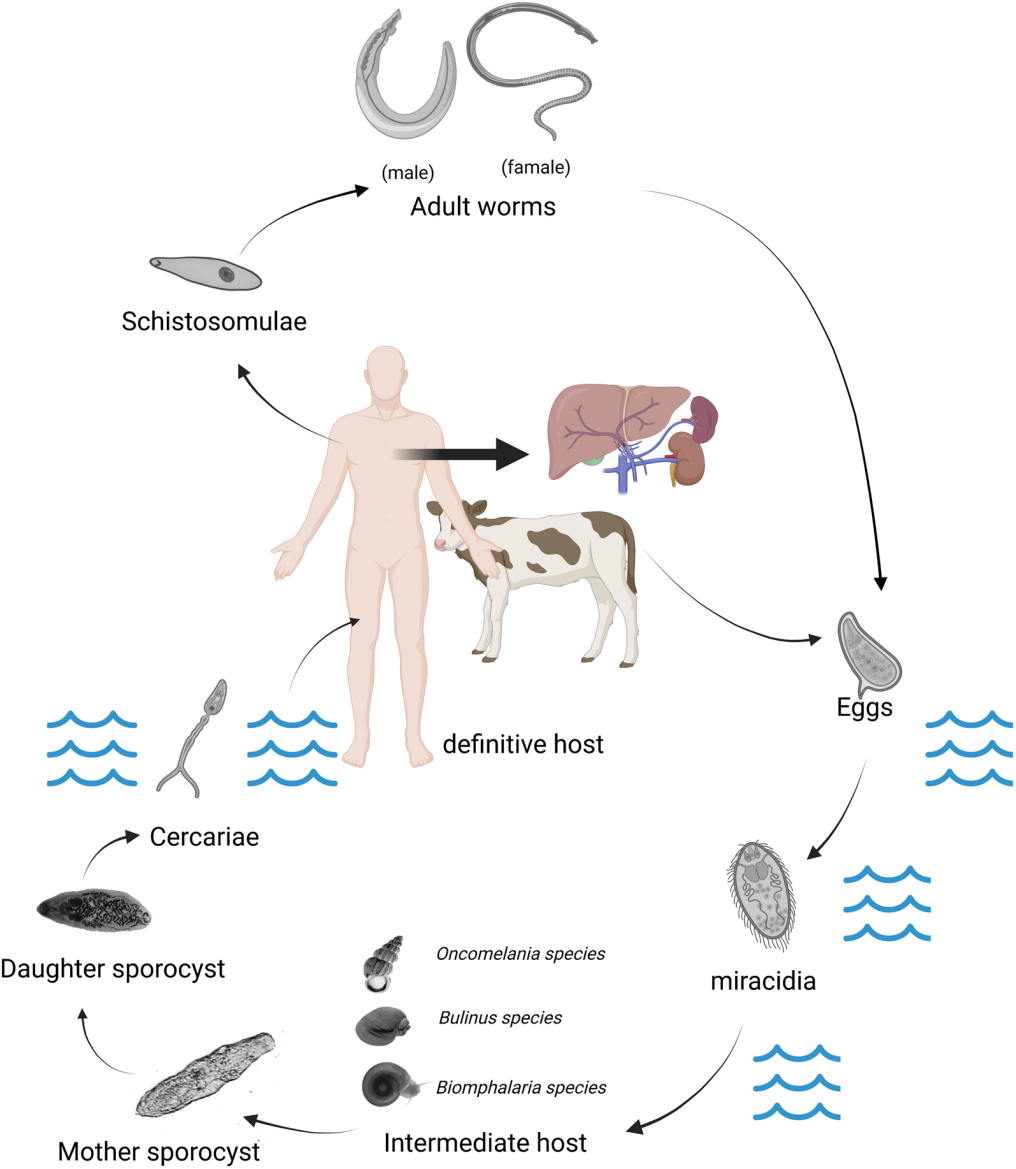
Take *Schistosoma japonicum* as an example to describe the life cycle. The life cycle of *S. japonicum* involves three distinct stages. (1) Eggs are released by adult *S. japonicum* flukes living in the veins of the host's intestine and are passed out of the host's body through feces. (2) The eggs hatch in freshwater and release miracidia, which swim to and infect specific snails, known as *Oncomelania* spp. These snails are the intermediate host of the parasite. (3) Inside the snail, the miracidia develop into sporocysts, which produce thousands of cercariae. These cercariae leave the snail and actively penetrate the skin of the host, usually a human or other mammal, swimming through the bloodstream to reach the host's liver, where they mature into adult flukes

The life cycle of these three blood flukes is highly similar (Fig. 1). They all require a specific gastropod freshwater or amphibious mollusk as an intermediate host to complete the larval development stage (asexual reproduction) before they can become the infective form (cercaria) for the final host mammals (including humans) [6].

In this article, we mainly discuss the relevant content of *S. japonicum*, so we focus on the disease of schistosomiasis *japonicum*, which belongs to the type of hepatointestinal schistosomiasis distributed widely in China, Japan, Indonesia, and Philippines. The transmission of schistosomiasis in endemic areas largely depends on the availability and abundance of suitable hosts, their susceptibility to parasite species, fecal egg count, time of egg excretion, and egg survival rate. According to these criteria, domesticated animals such as cattle, pigs, and dogs appear to be the most important animal hosts for the spread of schistosomiasis *japonicum* [7].

### Prevention and control of schistosomiasis *japonicum*

As the sole intermediate host of *S. japonicum*, the study of the morphological structure, life habits, distribution characteristics, and immune mechanisms of *Oncomelania* (genus) can control the number of snails without disrupting the ecological balance, thus achieving the goal of controlling the transmission of schistosomiasis [8]. Effective chemical molluscicides include Niclosamide, Pentachlorophenol, Nicotinamide, bromoacetamide, Trichlorfon, and Bromoacetamide. Niclosamide is currently the WHO-recommended commercial molluscicide, which is effective and has not shown resistance so far. However, its drawback is that it has some environmental toxicity [9]. Niclosamidate [10], novel salicylanilide ester derivatives [11], PBQ [1-(4-chlorophenyl)-3-(pyridin-3-yl)urea] [12], arylpyrrole [13], and other drugs are all new molluscicides under research today, which have shown good snail control efficacy under experimental conditions. In addition to the most widely used chemical snail control methods, physical and biological methods have also been used [14]. There are generally three types of biological control methods: animal based, plant based, and microorganism based [14, 15]. In biological control methods, there is an interesting phenomenon that the growth and development of *S. japonicum* are inhibited when the *Oncomelania hupensis* is infected with the harmless *Exorchis* sp. [16]. After being infected with *Exorchis* sp., the *O. hupensis* will produce a large immune response including blood lymphocytes and secretions. If the snail is subsequently infected with *S. japonicum* within 21–85 days, the previous immune response triggered by *Exorchis* sp. will severely interfere with the growth and development of *S. japonicum* larvae, leading to abnormal larval structure, growth arrest, and eventually death. However, these immune responses gradually disappear before the snail is reinfected with *Schistosoma*, which is not observed in snails infected solely with *S. japonicum* [16]. In addition to the three traditional molluscicidal methods mentioned above, with the advancement of research, many new molluscicidal methods have also been proposed, for example, controlling the reproduction of snails and then controlling the number of snails by studying their reproductive perspective [14]. From the perspective of repurposing existing drugs, aspirin is also a potential medication that can have an impact on *S. mansoni* parasitizing inside *Biomphalaria glabrata* [17].

Currently, praziquantel is the preferred drug for the treatment of all forms of schistosomiasis [18]. Laboratory has confirmed that *S. japonicum* can develop resistance under praziquantel pressure; since the exact anti-schistosomiasis mechanism of praziquantel is not clear, monitoring *Schistosome* resistance and exploring the molecular basis of resistance remain important tasks [19]. Currently, there is no commercial vaccine available for human schistosomiasis, but considerable progress has been made in vaccine development, with four vaccine candidates in various stages of human clinical trials [20]. As research on *S. mansoni* is relatively mature, the vaccine development of *S. mansoni* has provided a good inspiration for *S. japonicum*. For example, studies have found that *S. mansoni* lipid raft protein FLOTILLIN2 is a surface membrane protein with the potential to be a target for schistosomiasis vaccines. In this study, recombinant FLOTILLIN2 protein of *S. japonicum* was expressed in vitro by gene cloning, and sequence comparison and evolutionary tree analysis were carried out by bioinformatics methods, laying a foundation for further study on its potential as a vaccine [21, 22]. Due to the zoonotic nature of *S. japonicum*, animals (such as cattle, which act as intermediate hosts for up to 90% of the parasitic eggs in the environment [23]) are key reservoirs of the schistosomiasis transmission circle. From the perspective of vaccine research and development, it is necessary to study veterinary vaccines. Currently, many candidate vaccines for transmission blocking of *S. japonicum* have been developed [24].

While the strategies for the prevention and control of schistosomiasis are continuously evolving, currently, the main focus is on controlling the population and life cycle of the intermediate host. Globally, it remains challenging to eradicate schistosomiasis in the short term on a worldwide scale. To prevent and treat schistosomiasis, as well as to gain deeper insights into the intermediate host, research on the immune interactions between the parasitic blood fluke and the intermediate host snail is crucial. By comparing the well-studied immune responses of *B. glabrata* and *Bulinus truncatus* with the *O. hupensis*, we aim to provide direction for further in-depth research.

### Snail-associated immunological factors and mechanisms

As the intermediate host of *S. mansoni*, the immune system of *B. glabrata* has been extensively studied, and significant progress has been made in understanding the immune response elicited by *S. mansoni* infection [25–31]. The immune response of *B. glabrata* can be roughly divided into cellular and humoral immunity. Upon invasion, the pathogen-associated molecular pattern (PAMP) pattern recognition receptors (PRRs) on soluble or haemocyte surfaces recognize the *Schistosoma*, and haemocytes are recruited to the site of infection via chemoattractants such as MIF or other unknown mechanisms [32], leading to the so-called “encapsulation reaction”. Subsequently, haemocytes rapidly upregulate the expression of immune effector molecules and cytotoxic molecules (such as reactive nitrogen and oxygen species) by some unknown mechanism to kill the pathogen [28, 33]. On the other hand, some humoral factors such as fibrinogen-related proteins (FREPs) and thioester-containing proteins (TEPs) play a role in the initial pathogen recognition and can promote phagocytosis by binding to some unidentified receptors on haemocytes [33–35] as well as activate antimicrobial peptides or lytic factors (such as Biomphalysin) in the plasma to assist in killing and clearing the parasite [26, 33]. Cellular immunity is mainly carried out by haemocytes in haemolymph, while humoral immunity is mainly composed of various soluble immune factors, extracellular cytokines, chemokines, effector molecules, and cytotoxic molecules synthesized and secreted by haemocytes. The two work together to carry out an immune defense function against foreign pathogen invasion [33].

**Fig. 2:**
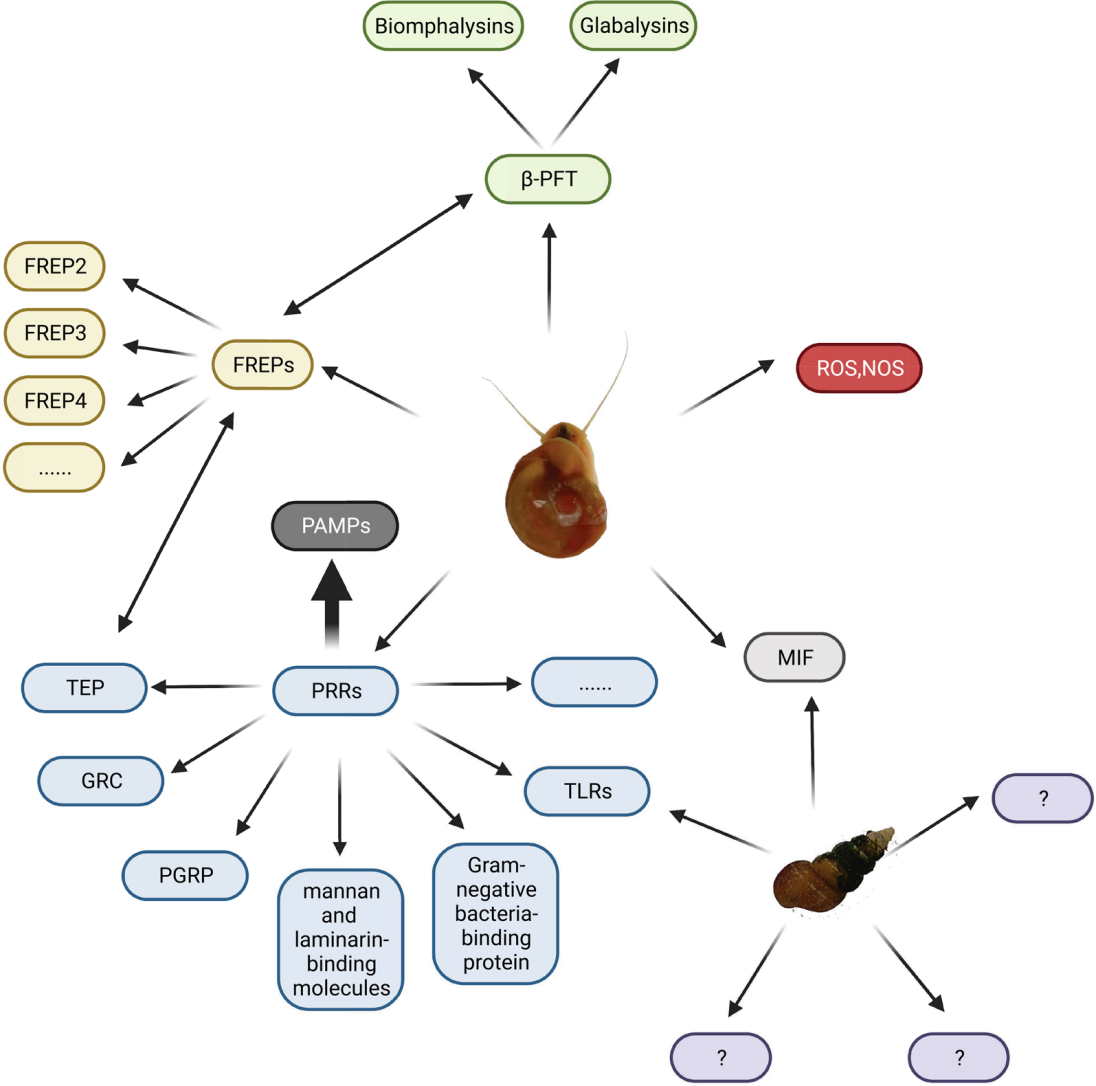
We summarized the important immune-related molecules of *Biomphalaria glabrata* and *Oncomelania hupensis* and expressed the interaction of immune-related molecules with bidirectional arrows. At the same time, it was found that there were still many gaps in immune-related molecules of *O. hupensis*

As a mollusk, *B. glabrata*, like many other invertebrates, protects itself from pathogen infection through its innate immune defense system [36]. Similar to *B. glabrata*, *O. hupensis* has two types of immune system: cellular and humoral [37]. Although research on the immune system of *O. hupensis* is relatively scarce, there has been a significant increase in recent years, and some research achievements have been made, which we will review later [37–42] (Fig. 2).

### Immune system of *Biomphalaria glabrata*

Overall, there are relatively few studies focused on the immune system of snails, with much of the current research being conducted on *S. mansoni* and its intermediate host, *B. glabrata*. *Biomphalaria glabrata* is a model mollusk with a sequenced immune cell genome, which has important implications for immunological research on mollusks and the practical prevention and treatment of schistosomiasis [43]. The immune defense system of *B. glabrata* against *S. mansoni* is an innate immune response, which involves the coordinated action of cellular and humoral immunity. Circulating haemocytes migrate to the site of *S. mansoni* miracidia and envelop them, and PRRs recognize PAMPs, activating multiple signaling pathways and immune effector molecules. Cell-toxic molecules such as reactive oxygen species (ROS) are released. The presence of dead cercariae not enveloped by haemocytes in drug-resistant individuals suggests the existence of humoral factors in their blood that can kill *S. mansoni* larvae [44].

Research on the immune mechanism of *S. mansoni* resistance in *B. glabrata* mainly focuses on three aspects: immune cells (haemocytes), humoral immune factors, and fibrinogen-related proteins (FREPs) [29]. The defense system of *B. glabrata* against *S. mansoni* is composed solely of innate immune components, which act in synergy between cellular and humoral immunity. Haemocytes are the main immune cells, and they are classified into granulocytes and transparent cells based on their size and granularity [45], with their proportion being closely related to the type of snail and varying with invasion by different pathogens [29]. Both transparent and granulocytes are associated with the phagocytic activity of *B. glabrata*, which is limited to the removal of the microvilli and small fragments of detached cercariae [46]. During phagocytosis, recognition of the monosaccharides released during the transformation of cercariae to schistosomula triggers rapid phagocytosis mediated by BgFREP3 [47]. Encapsulation is strongly associated with granulocytes [48], which are recruited to the site of invasion by miracidia, enveloping them with the aid of chemoattractants such as MIF and a potential attractant AIF [49]. The haemocytes involved in encapsulation can release cytotoxic substances to kill the schistosomes, the most important of which is ROS [50]. Potential cytotoxic substances include proteases and protease inhibitors [51]. Haemocytes may also directly react to the schistosomes [49]. Given the important role of haemocytes in the immune response against *S. mansoni*, it is crucial to study their proliferation and differentiation mechanisms. The known growth factor is granulocyte protein [27], and AIF is not only involved in the proliferation of haemocytes but also related to cell migration and encapsulation [52]. Of course, before regulating and controlling the signal pathway of immune response activation, immune recognition receptors involved in pathogen recognition include PRRs, integrin-related proteins, and growth factor/cytokine-like receptors. Among them, PRRs participate in the immune response against *S. mansoni*, including TLRs, guadrupe resistance complex (GRC), mannan and laminarin-binding molecules, peptidoglycan recognition proteins (PGRPs), gram-negative bacteria-binding proteins, lipopolysaccharide-binding proteins, and molecules with variable immunoglobulin and lectin domains [29]. In susceptible snail (M-line), it was observed that *S. mansoni*, after entering the host, releases an invadolysin called *S. mansoni* Leishmanolysin (SmLeish). SmLeish interferes with the chemotaxis of haemocytes, thereby affecting the host's immune response and increasing the success rate of *S. mansoni* infection. Simultaneously, experiments revealed that the chemotactic response of resistant snails (BS-90) is not influenced by SmLeish [53].

The *B. glabrata* is dependent on soluble immune factors in the haemolymph, mainly including PRRs (such as the thioester-containing protein TEP) and cytotoxins (biomphalysin, variable immunoglobulin, and molecules containing agglutinin domains) [29]. TEP is expressed by a subtype of haemocytes called blast-like cells and secreted into the haemolymph [54]. Eleven unique TEP transcripts are present in *B. glabrata*, and these TEP transcripts exhibit a high level of sequence identity at both the nucleotide and putative amino acid levels, regardless of whether it is a susceptible or resistant snail strain. However, there are differences in the baseline expression levels of several TEPs between the resistant (BS90) and susceptible (NMRI) snail strains, with C3-1, C3-3, and CD109 showing higher baseline expression levels in the resistant strain, while C3-2 and TEP-1 exhibit higher baseline expression levels in the susceptible strain [55]. TEP can interact with a special type of agglutinin called FREPs, recognize and form immune complexes with the variable surface glycoprotein SmPoMucs of the miracidia of *S. mansoni*, accelerate antigen dissolution, and promote the occurrence of immune reactions [44]. BgFREP2 is included in the starting complex of BgFREP3, which also contains BgTEP1. FREP3 plays a core role in resistance to the major snail pathogen (*S. mansoni*) [33]. FREPs are composed of N-terminal immunoglobulin superfamily (IgSF) domains that can be concatenated through intermediate regions (ICRs) and C-terminal fibrinogen (FBG) domains. Studies have found that both C-type lectin-related proteins (CREPs) and galactose-binding lectin-related proteins (GREPs) are also associated with immune responses against *Schistosoma* [56]. Biomphalysins belong to a type of β-pore-forming toxin (β-PFT) and is an important factor in humoral immunity that can directly lyse target cells [26].

**Fig. 3:**
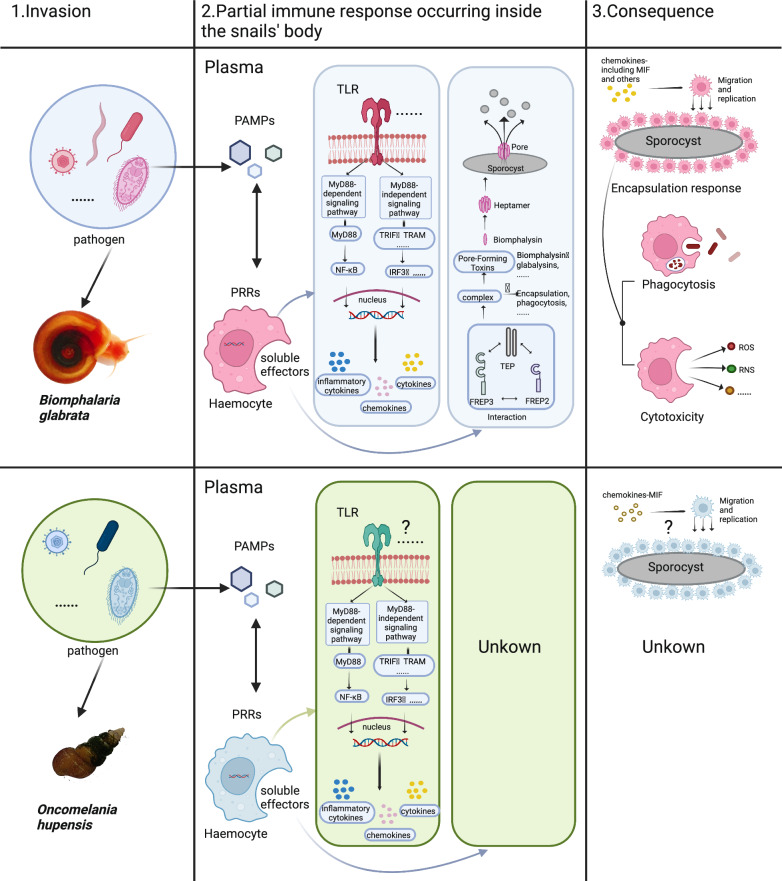
The upper panel of this figure illustrates the partial immune responses triggered by pathogen invasion in *Biomphalaria glabrata*. Several immune-related factors and their mechanisms of action have been reported in smooth-bore snails. BgTLR, as a crucial transmembrane PRR, mediates immune signaling in haemocytes through intracellular transduction. Soluble immune factors like BgFREP, BgTEP, and Biomphalysin interact to initiate cytotoxic effects (perforation of pathogen cell membranes or release of cytotoxic substances) and opsonization, culminating in pathogen elimination via cell phagocytosis. Additionally, the chemoattractant factor BgMIF induces haemocyte migration and proliferation, potentially contributing to encapsulation response. In contrast, the lower panel describes the immune response following pathogen invasion in *Oncomelania hupensis*. Research in this area remains relatively scarce. To date, only directly related immune factors, OhTLRs and OhMIF, have been identified and functionally characterized, with other immune-related factors and their associated mechanisms largely unknown

David Duval and others subsequently discovered a new β-PFT, glabalysin, but its specific immune mechanism, function, and role in immunological memory are still unknown, and we look forward to more in-depth research in the future [57]. Humoral components, especially FREPs and biomphalysins, are related to the innate immune memory of the *B. glabrata*, and experimental evidence has shown that the primary immune response is mainly cellular immunity, while the secondary immune response is humoral immunity [58]. These soluble immune effectors in the body can participate in directly killing the cercariae and can also be involved in preparing haemocytes to initiate cell-mediated immunity [29]. In contrast to resistant snails, the susceptible *B. glabrata*'s haemocytes cannot recognize the invading schistosomes, so they cannot be activated and do not generate subsequent immune responses. It is speculated that this situation is related to the polymorphic patterns of FREPs in *B. glabrata* and SmPoMucs in *S. mansoni* as well as the inhibition of haemocyte function by larval transformation products (LTPs) [59]. Proteomics has also identified binding proteins between the cercariae and proteins that are not usually related to immunity and defense, such as actin, collagen, haemoglobin, GAPDH, lipoprotein, and histone 4, and the binding of these "non-immune" proteins to the cercariae is specific. They may also have some undocumented immune effects [44]. The immune responsiveness of the *B. glabrata* is also affected by external stressors, such as temperature: when maintained at 32 °C, the BS-90 snails, which are resistant to *S. mansoni* at 25 °C, become susceptible in the F2 generation, indicating epigenetic inheritance. Recent research has found that this is related to the silencing of PIWI-encoded transcripts, as PIWI inhibits the expression of retrotransposons such as nimbus. When nimbus expression increases, resistant snails become sensitive [60]. Heat shock increases the mRNA level of NADPH oxidase 2 and hydrogen peroxide produced by snail haemocytes, and an HSP-90 inhibitor can reverse both phenotypes [61]. The change in temperature involved, where higher temperatures make snails more susceptible, suggests that with global warming, the spread of schistosomiasis is likely to become easier, so vigilance in prevention and control is essential. Discovering new foci may contain new solutions to the problem of schistosomiasis, and in recent years, research on the microbiome of *B. glabrata* may be a promising angle [54] (Fig. 3).

### Immune system of *Bulinus truncatus*

In 2018, the genome sequence of *B. truncatus*, the intermediate host of *S. haematobium*, was obtained [62]. Andreas J. Stroehlein et al. assembled this transcriptome from short- and long-read RNA sequencing data, predicting 12,998 proteins, with 58% of them having homologues in *B. glabrata* [63]. The genome of *B. truncatus* contains multiple gene families related to parasite infection and immune response, including but not limited TLR, Down syndrome cell adhesion molecules (Dscam), lysozyme, C-type lectins, and immune-associated molecules such as cytokines and chemokines. The existence of these gene families not only reveals the immune response mechanism of *B. truncatus* but also provides new clues for future research on its interaction with *S. haematobium* [62]. Based on the current literature, the main focus of research on the immune response of *B. truncates* is haemoglobin-related protein (HPR) and antimicrobial peptides. Both *B. truncatus* and *B. glabrata* can resist schistosomes through innate and adaptive immune responses. The immune-related genes involved in both include TLR, Dscam, lysozymes, C-type lectins, etc., but there are differences in their immune response mechanisms. *Biomphalaria glabrata* has multiple immune molecules, mainly involving HPR and antimicrobial peptides, while the immune response of *B. truncatus* mainly involves its unique HPR and BtPMAP antimicrobial peptides. In addition, their epigenetic regulatory mechanisms may also be different. *Biomphalaria glabrata* can regulate the expression of related genes through epigenetic modifications such as DNA methylation and histone modification when infected with schistosomes, while the epigenetic regulatory mechanism of *B. truncatus* is currently unclear. Due to the limited research on the immune response of *B. truncatus*, further in-depth research is needed to compare it with *B. glabrata* [64].

### Cellular immunity of *Oncomelania hupensis*

Haemocytes are the main effector cells of innate immune defense, playing a crucial role in the host defense against parasitic infections. Without the encapsulation reaction, cytotoxicity, and phagocytosis driven by haemocytes, parasites can survive and establish infection within the snail.

To date, there is no consensus on the classification of haemocytes in *O. hupensis* as different studies propose different classification systems [37, 39, 65, 66]. For instance, Xu et al. [65] classified the haemocytes in *O. hupensis* into granular round cells, agranular round cells, and spindle-shaped cells, while another research team categorized them into round cells with filopodia, acidophilic round cells without filopodia, alkaline round cells without filopodia, and spindle-shaped cells [67]. Typically, the haemocytes of most gastropods are classified into granulocytes and agranulocytes based on their morphology, and *O. hupensis* are no exception, with different proportions and subgroups among species [37]. Granulocytes are a minority group among all haemocytes in *O. hupensis*, accounting for < 10% of the total population. They are mostly round shaped, ranging in size from 4.3 to 10.9 μm, with a few being spindle-shaped. Further classification based on granule properties divides granulocytes into acidophilic granulocytes and alkaline granulocytes, as observed by Giemsa staining and electron microscopy [37]. Agranulocytes, or transparent cells, make up > 90% of all haemocytes and vary in size from 0.4 to 30.8 μm [37]. Based on their size, they can be classified into small, medium, and large transparent cells, with decreasing proportions in that order [37]. Transparent cells can also be classified based on their shape as round, elliptical, and pseudopodia-like cells [37].

The number and type of haemocytes are essential cellular immune elements for establishing a successful immune response, although it seems that they do not determine the effectiveness of encapsulation [28, 29]. When a healthy, uninfected snail is attacked by *S. japonicum* cercariae, the number of haemocytes increases immediately and reaches its peak in 6 h, approximately twice the number before infection. The number of haemocytes then gradually decreases, significantly lower than the pre-infection level 12 h after infection, and reaches its lowest point 24 h after infection. The number of haemocytes then slowly increases, remaining lower than the pre-infection level on the 8th day after infection [37]. Current research suggests that large transparent cells and granulocytes play a dominant role in the early defense response of snails. However, the differentiation, migration, and action mechanisms of granulocytes and transparent cells in snail haemocytes require further investigation [37].

### Humoral immunity of *Oncomelania hupensis*

From the currently published articles, research on the factors and mechanisms involved in the immune interaction between *O. hupensis* and the *S. japonicum* is very limited. In recent years, some research progress has been made, and some immune factors and effector factors have been identified, including MIF [39, 41], Toll-like receptor (TLR) [42], myeloid differentiation factor 88 (MyD88) [42, 68], and thioredoxin (Trx) [40, 69]. These immune and effector factors have been identified and functionally studied, involving stimulating cell proliferation, activating immune signaling pathways, regulating the release of toxic molecules, and increasing resistance to schistosome infection.

MIF was first discovered in the human body in 1966. Research has found that it plays a role in delayed hypersensitivity reactions and is a soluble cytokine that effectively inhibits macrophage migration in the body, hence its name [70]. Macrophage migration inhibitory factor is an evolutionarily conserved immune protein that is widely expressed in the biological world. In mammals, MIF has about 90% homology [71]. In humans, mice, and rats, a mRNA of about 0.8 kb is encoded, which encodes a non-glycosylated protein of 114 amino acids with a relative molecular weight of 12.5 kDa [72]. In the human body, there is only one MIF gene located on chromosome 22 (22q11.2), which consists of three short exons of 107, 172, and 66 base pairs and two introns of 188 and 94 base pairs, respectively [72]. According to X-ray crystallography studies, MIF is a homotrimer, with each monomer consisting of 115 amino acids. In each monomer, two α-helices are filled in a four-stranded β-sheet layer, and three β-sheet layers and six α-helices form a circular protein. The trimer center forms an internal passage [73]. In addition to its cytokine functions, MIF plays a hormone-like role in regulating blood sugar and glucocorticoids [71]. Macrophage migration inhibitory factor was originally found to regulate the activity of T cells in acquired immunity, and subsequent studies have found that it plays a key role as a regulator of innate immunity. Macrophage migration inhibitory factor is a pleiotropic inflammatory mediator that serves as a biomarker for various diseases and is associated with the pathogenesis of sepsis, inflammation, and autoimmune diseases [71]. Macrophage migration inhibitory factor promotes the proliferation of immune cells and inhibits apoptosis via the classical receptor-mediated pathway or through non-classical endocytic pathways [72]. It regulates the upregulation of TLR4 expression on intracellular MIF, which is a signaling molecule on macrophages that responds to the gram-negative bacterial endotoxin receptor complex. Various pathogens and other proinflammatory cytokines can induce the release of MIF in macrophages. Macrophage migration inhibitory factor plays a regulatory role in innate and adaptive immune responses and is an important component of the host's anti-pathogen alarm system and stress response [72].

In 2010, the MIF of the *B. glabrata* (BgMIF) was identified for the first time, possibly the first functional endogenous cytokine in gastropods [32, 39]. It participates in the immune response of snails, stimulates cell proliferation, and inhibits NO-induced cell apoptosis. In vitro experiments have shown that BgMIF can promote encapsulation of *S. mansoni* larvae. Knockdown of BgMIF in vivo prevented the changes in circulating haemocyte populations that occur in response to *S. mansoni* infection and led to a significant increase in snail burden, partly determining the compatibility between the parasite and the snail [39].

Huang et al. confirmed through experiments that the *O. hupensis* macrophage migration inhibitory factor (OhMIF) is expressed in various tissues of the snail, especially in immune cell types such as haemocytes and is localized in the cytoplasm. When the snail is infected by schistosomes, the expression level of OhMIF is significantly upregulated. Knocking down the expression of OhMIF in the snail significantly reduces the proportion of phagocytic haemolymph in the total circulating haemolymph as well as the proportion of larger volume and higher particle density haemolymph, indicating that OhMIF not only participates in the activation and differentiation of haemocytes but also plays an important role in promoting the migration and recruitment of haemocytes to the site of infection [39]. By determining the crystal structure of OhMIF, it was found that OhMIF consists of four monomers from an asymmetric unit, with three monomers forming a homologous trimer and the fourth monomer forming a trimer with two other monomers from adjacent asymmetric units [74]. *Oncomelania hupensis* macrophage migration inhibitory factor has the same fold as the human MIF (hMIF) monomer but has a structure that other MIFs do not have, namely a long C-terminal helix (Hα3), which maintains thermal stability and activates tautomerase, but is not necessary for the activation of the ERK1/2 pathway [74]. By site-directed mutagenesis, glycine was substituted for proline 2 at the N-terminus of OhMIF, and a mutant strain rOhMIFP2G was successfully expressed and purified. No tautomerase activity was detected in rOhMIFP2G, indicating that rOhMIF displays a conserved D-dopachrome tautomerase activity dependent on Pro2, which can be significantly inhibited by the MIF antagonist ISO-1. rOhMIF and its mutant rOhMIFP2G can also induce phosphorylation and activation of the ERK1/2 pathway in circulating haemocytes, indicating that tautomerase activity is not necessary for the activation of the ERK1/2 pathway [41]. It is known that OhMIF plays an important role in the immune response of *O. hupensis* to resist schistosomiasis infection, but its signaling functions in cell proliferation, apoptosis, and survival remain to be studied [41].

As early as 1988, when Hashimoto et al. studied the embryonic development of *Drosophila*, they found that there was a gene (dToll) that determined the dorsoventral differentiation of *Drosophila*, and the transmembrane receptor protein it encoded was called Toll receptor [75]. In 1997, the first Toll-like protein was found on the surface of human cells, and it plays an important role in human immunity [76]. To date, the presence of TLRs has been found in lower plants and lower animals except *Drosophila* and humans [77]. Toll-like receptors are transmembrane proteins (all currently found as type I transmembrane proteins) consisting of three parts: leucine-rich repeats (LRRs) extracellular regions, transmembrane segments, and cytoplasmic regions containing the Toll/IL-1 receptor homologous region (TIR) region responsible for signal transduction and activation effector functions [78]. The TLR is thought to be PRRs, and PAMPs bind to PRRs to initiate an immune response [79]. In the Toll-like signaling pathway, MyD88 containing the TIR domain is a typical linker protein, and MyD88 connects IL-1 receptor (IL-1R) or TLR family members to IL-1R-associated kinase (IRAK) family kinases through homotypin-protein interactions, a process that is associated with nuclear factor κB (NFkB). Activation of mitogen-activating protein kinases and activator protein-1 is associated [80]. Obviously, the intracellular linker protein, known besides MyD88, includes TIRAP, TRIF, TRAM, etc.; according to the difference of linker protein, the signaling pathway of TLRs can be divided into MyD88-dependent signaling pathway and MyD88-independent signaling pathway [77]. TLRs are major recognition receptors in innate immune responses [81] and, in acquired immunity, are able to recognize microbial components that activate dendritic cells (DCs) [82].

A study in 2016 provided the first functional characterization of BgTLR in *B. glabrata* [31]. Toll-like receptors play a critical role in innate immune responses by directly recognizing pathogens (typically bacteria, viruses, and fungi) or by binding to endogenous ligands that recognize pathogens and transmitting signals to immune cells [31, 83]. BgTLR has complete LRR and TIR domains and is involved in the immune response of the *B. glabrata* to *S. mansoni*. Here, we report the first functional report of a snail TLR and demonstrate its essential role in the cellular immune response of *B. glabrata* following a challenge with *S. mansoni*. Two subspecies of *B. glabrata* with different *S. mansoni* compatibility phenotypes were studied. The resistant strain (BS-90) showed higher levels of BgTLR than the susceptible M-line strain. Following a challenge with *S. mansoni*, the transcriptional expression of BgTLR was rapidly upregulated in the resistant BS-90 snails, while it did not increase significantly in susceptible M-line snails. Knockdown of BgTLR using targeted siRNA oligonucleotides resulted in a significant change in the resistance phenotype in resistant snails, with approximately 40% of normally resistant snails becoming infected. These results demonstrate that BgTLR is a critical snail immune receptor that can influence the partially determined resistance phenotype of *B. glabrata* to *S. mansoni* [31].

Zhao et al. identified 16 TLRs in *O. hupensis*. *Oncomelania hupensis* Toll-like receptors were highly expressed in the haemocytes of snails, and the expression of nine OhTLRs in the gonads of female snails was higher than that of other tissues, and it was speculated that there may be maternal immune transfer in *O. hupensis*, while only the expression of OhTLR12 in gonads was observed in male snails compared with other tissues [42]. When snails are infected with schistosomiasis, the expression levels of all OhTLRs are significantly upregulated at 6 h, and in haemocytes, many OhTLR expression levels are inhibited at later time points, while in other tissues they are inhibited and fluctuate to varying degrees. The OhMyD88 gene was also highly expressed in haemocytes, and the expression of OhMyD88 in the whole snail was rapidly upregulated at 6 h. At 12 h, the levels of OhMyD88-1, OhMyD88-2, and OhMyD88-3 reached their highest values, respectively [42]. At 24–96 h, OhMyD88-1 dropped to normal, OhMyD88-2 and OhMyD88-3 increased moderately, and then the time point decreased and returned to normal [42]. As a downstream linker protein of the TLR signaling pathway, MyD88 is closely related to the dynamic changes between the two, and researchers have shown that TLRs are not only involved in the innate immune response of *O. hupensis* against the early response of *S. japonicum* but also speculate that they may play a role in the activation of different haemocytes [42].

Many studies have shown that cytotoxic molecules such as ROS and RNS are crucial for killing invading parasites in *S. mansoni* and support haemocyte-mediated damage and killing of miracidia of *S. mansoni* [33, 84]. Conversely, *S. mansoni* cercariae may protect themselves from harmful oxidative environments in the host during the early stages of infection through some redox systems, such as glutathione (GSH) and Trx [85]. Therefore, pathways and molecules involved in ROS production and clearance will affect the immune defense outcome in snails. Studies in humans have demonstrated that Trxs are a group of small-molecule proteins widely present in all living cells, and are critical regulators of cellular redox homeostasis.

Thioredoxin was first reported as an electron donor for *Escherichia coli* ribonucleic acid reductase in 1964 [86]. Thioredoxin is a small protein (molecular weight of about 12 kDa) widely present in prokaryotes and eukaryotes: *E. coli* contains two soluble Trx; yeast contains two soluble Trx and one mitochondria-specific Trx; Trx is present in the cytoplasm, chloroplast, mitochondria, and nucleus of plants. Only three widely expressed Trxs are found in the human body, Trx 1, Trx 2, and TXL 1 [87]. The unique folding pattern of TRX is named TRX folding, which consists of a single domain with a central five-stranded β-sheet with four flanking α-helixes and a dithiol/disulphide group in the active site forming a compact spherical structure with a highly conserved active central site between β2 and α2-Cys-Gly-Pro-Cys-Cys-(-CGPC-) [87]. Thioredoxin has two forms of existence, oxidation state and reduced state, and can participate in redox reactions because the two cysteine sulfhydryl groups in the active site can reversibly form disulphide bonds [88]. Thioredoxin is involved in many physiological processes and has a variety of biological functions, including redox regulation, signaling, regulation of transcription factors (such as NF-κB, Ref-1-dependent AP1, etc.), DNA-binding activity, and participation in the regulation of cell growth and apoptosis [88]. In humans, defects in folding are associated with the onset of a variety of diseases, such as cancer, Alzheimer's disease, and cystic fibrosis [89]. A protein containing an active site motif (-CXXC-) with two cysteines (X can be any amino acid) called thioredoxin-related protein (TRP) belongs to the Trx supergene family, which includes thiotransferases, eukaryotic proteins belonging to the protein disulphide isomerase (PDI) family, and some bacterial proteins [90]. As a member of the Trx superfamily, 14 kDa of human Trx-associated protein (TRP14) was originally found from human hella cells, containing five cysteines (Cys43, Cys46, Cy s64, Cy s69, and Cys110); only two cysteines (Cys43 and Cys46) formed the active site of CXXC [-Cys-Pro-Asp-Cys-(-CPDC-)], a structure related to its redox activity [91].

Cao et al. identified TRP14 in *O. hupensis* and explored whether OhTRP14 participates in the clearance and regulation of ROS in circulating haemocytes in *O. hupensis* in response to *S. japonicum* [40]. OhTRP14 is expressed in all tissues and haemocytes of snails; when the snails are infected by schistosomiasis, the expression of OhTRP14 in snails shows obvious upregulation, and the level of ROS in circulating haemocytes is also significantly increased. If the expression of OhTRP14 in the snails is knocked down, the level of ROS in the circulating haemocytes of infected snails is significantly increased [40]. The Cys41 (TGC) residue located in the motif of the active site of CPDC was replaced with Ser (AGC). Mutant (rOhTRP14C41S) and rOhTRP14 were expressed in *E. coli*, the enzymatic activity of both proteins was studied by insulin disulphide reduction assay. rOhTRP14 showed significant oxidoreductase activity, and the mutant rOhTRP14C41S did not detect enzyme activity under the same assay conditions. It shows that rOhTRP14 conserved oxidoreductase activity is dependent on the CPDC motif [40]. The specific mechanism of OhTRP14 in *O. hupensis* needs to be further studied.

Although TRP has been identified and functionally characterized in *O. hupensis*, there are no literature reports on TRP in *B. glabrata*. This provides a new research perspective for studying the redox balance and ROS-related mechanisms in *B. glabrata*.

## Conclusions

As a model organism, *B. glabrata* will be studied in depth, and it can be an inspiration regarding other organisms. We can validate and study the immune factors already identified in *B. glabrata* on the less studied *O. hupensis*, which saves both time and resources. In this article, we reviewed the basic knowledge of *O. hupensis* and *S. japonicum* and summarized the three main immune factors studied in recent years in immunology of the *O. hupensis*: MIF, Trx, and TLR. At the same time, we compared the model organism *B. glabrata* with the hot research organism *B. truncatus*, trying to find inspiration in the more thoroughly studied *B. glabrata*, to study the immunology of the *O. hupensis*, *B. truncatus*, invertebrates, and even mammals. We believe that with the deepening of research on the intermediate hosts of schistosomiasis, control of the spread of schistosomiasis is imminent. We have more confidence and ability to achieve the anticipated goal of eliminating schistosomiasis by 2025.

## Data Availability

This article is a review paper and does not generate or analyze any new datasets. All referenced data and information are sourced from publicly available literature and materials. These referenced data points originate from the primary sources of their respective studies, accessible through their corresponding original publications. Readers are encouraged to refer directly to the cited literature for further details and to ensure the accuracy and completeness of the data.

## Abbreviations

*O. hupensis*: *Oncomelania hupensis*
*B. glabrata*: *Biomphalaria glabrata*
*S. japonicum*: *Schistosoma japonicum*
*S. mansoni*: *Schistosoma mansoni*
*S. haematobium*: *Schistosoma haematobium*
MIF: Macrophage migration inhibitory factor
TLRs: Toll-like receptors
Trx: Thioredoxin
MyD88: Myeloid differentiation factor 88
